# The Endocannabinoid System and Cannabidiol: Past, Present, and Prospective for Cardiovascular Diseases

**DOI:** 10.3390/ph14090936

**Published:** 2021-09-17

**Authors:** Martina Rabino, Sara Mallia, Elisa Castiglioni, Davide Rovina, Giulio Pompilio, Aoife Gowran

**Affiliations:** 1 Unit of Vascular Biology and Regenerative Medicine, Centro Cardiologico Monzino-IRCCS, 20138 Milan, Italy; martina.rabino@cardiologicomonzino.it (M.R.); sara.mallia@cardiologicomonzino.it (S.M.); elisa.castiglioni3@gmail.com (E.C.); davide.rovina@cardiologicomonzino.it (D.R.); giulio.pompilio@cardiologicomonzino.it (G.P.); 2 Department of Cardiac Surgery, Centro Cardiologico Monzino-IRCCS, 20138 Milan, Italy; 3 Department of Biomedical, Surgical and Dental Sciences, University of Milan, 20138 Milan, Italy

**Keywords:** endocannabinoid system, endocannabinoids, cannabinoid receptors, phyto-cannabinoids, synthetic cannabinoids, cardiomyopathy

## Abstract

In the past, cannabis was commonly associated with mysticism and illegality. Fortunately, in recent years perspectives and discourses have changed. More prominence has been given to the rigorous scientific effort that led to the discovery of cannabis’ many physiological actions and endogenous signalling mechanisms. The endocannabinoid system is a complex and heterogeneous pro-homeostatic network comprising different receptors with several endogenous ligands, numerous metabolic enzymes and regulatory proteins. Therefore, it is not surprising that alterations and dysfunctions of the endocannabinoid system are observed in almost every category of disease. Such high degree of pathophysiological involvement suggests the endocannabinoid system is a promising therapeutic target and prompted the translation of resurgent scientific findings into clinical therapies. Shifting attitudes toward cannabis also raised other matters such as increased patient awareness, prescription requests, self-medication, recreational use, recognition of new knowledge gaps, renewed scientific activity, and seemingly exponential growth of the cannabis industry. This review, following a general overview of cannabis and the endocannabinoid system, assiduously describes its role within the context of cardiovascular diseases, paying particular attention to the Janus influence that endocannabinoid system modulators can have on the cardiovascular system.

## 1. Introduction

The main objective response of the human body to smoked cannabis is a marked increase in heart rate (HR). Moreover, the risk of acute myocardial infarction (MI) is raised during the hours following cannabis consumption. On the other hand, many countries have authorized cannabis use for medical conditions such as chronic pain, multiple sclerosis, cachexia, nausea, anxiety, and rare forms of paediatric epilepsy, while others have fully legalized the supply and use of cannabis. With this review we aim to contribute engaging material that is perhaps novel for some readers.

The cannabis plant belongs to the family *Cannabaceae* that has two main species *Cannabis sativa* and *Cannabis indica*. The plant and its many preparations have a history of recreational use, as well as medicinal and spiritual practices dating back thousands of years. These usages were well known and exploited in China, Egypt, Assyria, and India where cannabis was a highly traded commodity. The assigned Assyrian names “azalla” (connected to the term “azallu”, meaning to spin) and “gan-zi-gun-nu” (meaning the drug that takes away the mind) illustrate that the duality of its activity was early appreciated and utilized [1]. By the 19th century, chemists had isolated other plant-based active molecules (e.g., the alkaloids such as atropine from *Atropa belladonna*, muscarine from *Amanita muscaria* and morphine from *Papaver somniferum*). When a similar approach was taken to isolate active cannabis constituents, the results were not successful, because compared to the previously mentioned water-soluble plant alkaloids, components of the cannabis plant require organic solvents for isolation [2]. Nevertheless, by the latter half of the 20th century, delta-9-tetrahydrocannabinol (Δ^9^-THC) and cannabidiol (CBD) were among the first purified and structurally identified plant cannabinoids [3]. In the 1980s and 1990s the cloning and functional activity of two types of cannabinoid receptors (CBRs) were described [4,5]. This prompted the discovery of their endogenous ligands, the endogenous cannabinoids (eCBs), which were first isolated from brain tissue. In addition, the metabolic apparatus of the ECS was also starting to be determined [6,7]. It is now recognized that CBRs, eCBs and their metabolic enzymes are functional in both central and peripheral tissues; e.g., within the cardiovascular system CBR activity and eCB metabolism, they have been reported in cardiomyocytes, cardiac fibroblasts, vascular endothelial and smooth muscle cells, cardiac vagal afferent neurons, peripheral/resident immune cells, and platelets [8,9,10].

Equipped with this background information, the review will now set out to deepen the reader’s knowledge by presenting the discovery of endogenous cannabinoid signalling, its role in several cardiovascular diseases (CVDs), and attempts to harness its activity for therapeutic gains.

## 2. The Endogenous Cannabinoid System

The endogenous cannabinoid system (ECS) is an intricate and heterogeneous signalling network comprised of (i) eCBs such as N-arachidonoyl-ethanolamine (also known as anandamide which stems from the Sanskrit word “ananda” meaning bliss and its ethanolamide chemical structure), 2-arachidonoylglycerol (2-AG), and other cannabimimetic ligands, e.g., oleoylethanolamide, stearoylethanolamine; (ii) G protein-coupled receptors, CB1 and CB2; (iii) metabolic enzymes, e.g., fatty acid amide hydrolase (FAAH) and monoacylglycerol lipase (MAGL) and other proteins that regulate eCB tissue concentrations and/or cellular distribution [11]. The interaction between these components governs the normal level of activity of the ECS, referred to as ECS tone, which plays a vital role in diverse pathophysiological mechanisms spanning pre-conception to age- or disease-related decline in function.

### 2.1. Endocannabinoid Metabolism

**Figure 1:**
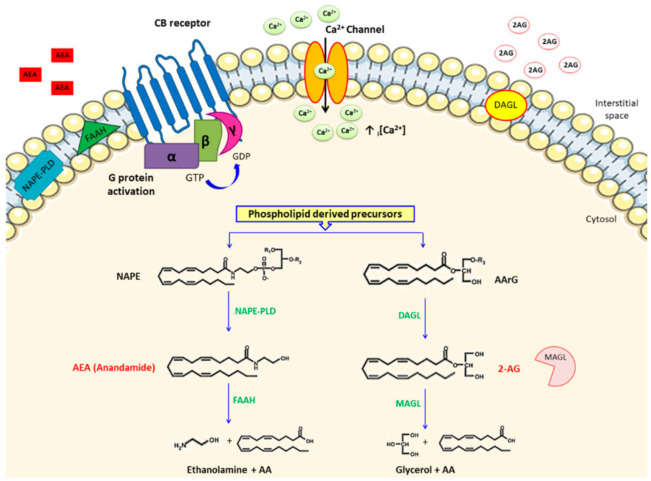
A schematic summary of the main endocannabinoid metabolic pathways. The biosynthesis of the two most researched eCBs, arachidonoyl ethanolamide (AEA) and 2-arachidonoylglycerol (2-AG), starts with the catabolism of phospholipid precursors via the action of two respective enzymes, namely, NAPE phospholipase D (NAPE-PLD) and the alpha or beta isoform of diacylglycerol lipase (DAGL). Despite AEA and 2-AG both possessing an AA chain structural motif, their catabolic pathways are different, and thus are respectively catabolized by fatty acid amide hydrolase (FAAH) and monoacylglycerol lipase (MAGL) to form arachidonic acid (AA) and ethanolamine following AEA degradation, or AA and glycerol in the case of 2-AG breakdown. Both products are rapidly re-incorporated into membrane phospholipids. Blue arrows signify enzymatic reactions; black arrow indicates the passage of ions through the ion channel; ↑ _i_[Ca^2+^] represents elevated intracellular calcium.

The first eCBs identified, and the most characterized, were the arachidonic acid (AA) derivatives anandamide (AEA) and 2-AG. eCB biosynthesis occurs on demand mostly following elevated intracellular calcium (_i_[Ca^2+^]) and the activity of multiple biosynthetic pathways (Figure 1). AEA is mainly synthesized by the hydrolytic conversion of the phospholipid hormone precursor N-acyl-phosphatidylethanolamine (NAPE) mediated by the NAPE phospholipase D (NAPE-PLD) enzyme. In addition to this canonical pathway, studies have shown that there were other possible pathways for AEA biosynthesis. For instance, an unidentified phospholipase C (PLC)-like enzyme was postulated to form AEA via hydrolyzing NAPE. Alternatively, NAPE acyl groups (sn-1 and sn-2) can be cleaved by alpha-/beta-hydrolase domain type 4, followed by hydrolysis of glycerophospodiesterase-E1. Lastly, another potential biosynthetic route for AEA could involve the action of a soluble form of phospholipase-A2 followed cleavage by lyso-phospolipase-D [6]. Instead, 2-AG is synthesized almost exclusively by the alpha isoform of diacylglycerol lipase (DAGL alpha) in the brain, and in the periphery by the beta isoform (DAGL beta), although exceptions to this rule were reported [12,13,14]. In both cases the DAGL alpha/beta lipases catalyse the hydrolysis of diacylglycerol (DAG), releasing a free fatty acid and 2-AG [13]. DAGs, the pre-cursors of 2-AG, are themselves mostly produced from the hydrolysis of sn-2-arachidonoyl-phosphatidylinositol-4,5-bisphophate (PIP2) species by the PIP2-selective PLC beta isoform [15].

Both AEA and 2-AG are characterized by a short in vivo half-life due to a two-step process: membrane trafficking followed by catabolic enzyme metabolism. The regulatory mechanism(s) of the bi-directional movement of eCBs across the plasma membrane (PM) is still controversial. One straightforward suggestion involved simple PM diffusion driven by an inward concentration gradient formed by intracellular enzymatic degradation. However, this is unlikely considering the development of synthetic selective uptake inhibitors [16]. The identification of several cytosolic AEA “chaperone” proteins, e.g., fatty acid binding proteins, heat shock protein 70, and a FAAH-like AEA transporter raised the possibility that PM diffusion could be followed by carrier-mediated intracellular transport to effector proteins, catabolic enzymes, or sequestration sites [16,17]. To provide an integrative model, it was proposed that AEA accumulates in specific PM domains where it could bind specific transporter proteins that translocate AEA across the lipid bilayer and/or present AEA to intracellular shuttles for transport to target/degradation sites [18]. Rimmermann et al. characterized the compartmentalization of the ECS and determined that 2-AG was concentrated in lipid rafts (LRs), co-localized with DAGL alpha and an arachidonoyl-containing DAG, while endogenous AEA and NAPE-PLD mirrored one another in both LRs and non-LRs [19]. Another means of regulating eCB availability was proposed to involve caveolae/LR-related endocytosis [20]. Caveolae are a special types of LRs that form isolated invaginated PM micro-domains comprising proteins such as caveolins, ion channels, and sequestered bioactive lipids. Caveolae are involved in several processes, e.g., receptor and ion channel function, mechanical stability, signal transduction, mitochondrial function, and cholesterol trafficking. Further investigation [19,21] on the involvement of caveolae in ECS tone determined that caveolae were discrete sites of AEA production and activation of CB1 receptor signalling. It was also observed that treatment with AEA caused CB1 translocation from caveolae, which is suggestive of agonist-induced modification. Therefore, besides representing a favourable platform to regulate AEA-CB1 signalling, caveolae might also be a cellular device for CB1 intracellular trafficking [22]. The association of caveolae/LR with sequestering eCBs holds particular significance for the heart and CVDs since caveolae and associated proteins, e.g., caveolin 3, play essential roles in cardiomyocyte signalling, mitochondrial function, and mechanical stability [23,24,25,26].

### 2.2. Cannabinoid Receptors

CB1 is mostly expressed in the brain with particularly high levels in the hippocampus, cerebellum, and basal ganglia. However, CB1 is also expressed in other sites including peripheral sensory neurons, the immune system, gastrointestinal tract, reproductive organs, adipose tissue, liver, skeletal muscle, pancreas, and cardiovascular tissues [27]. CB2 was initially identified in immune tissue and later also identified in the brain [28]. It is still unclear if CBRs function under physiological conditions or solely play a role in certain pathologies. For instance, a close interaction between ageing and sex was shown for the presence and distribution of CB1 and CB2 in the heart, with a preferential location of both receptors at cardiomyocyte intercalated discs and cytoplasm in the hearts of subjects older than 50-years-old, and decreased CBR expression in women under 50-years-old [29,30,31,32,33].

### 2.3. Beyond CBRs

In 1999 the first evidence for the existence of CBRs other than CB1 and CB2 was uncovered during a study on the vasodilator effect of AEA in preparations of perfused isolated rat mesenteric arterial beds. The study showed AEA elicited long-lasting vasodilatation and treatment with specific synthetic CB1 and/or CB2 agonists inhibited the dilatory effect of AEA. As a result, the existence of an endothelial receptor distinct from CB1 and CB2 was proposed to cause AEA-induced mesenteric vasodilation, which was later called the abnormal-CBD receptor [34]. Likewise, Zygmunt et al. used AEA-induced vasodilation to uncover the interaction between AEA and the transient receptor potential vanilloid type 1 (TRPV1) channel [35]. TRPV1 are nonselective cation channels belonging to the transient receptor potential (TRP) channel family that integrate multiple noxious stimuli, e.g., heat (>42 °C), low pH (<6.0), or capsaicin (the constituent in pungent chili peppers). Another member of the TRPV subfamily, TRPV4, correlated with AEA vasorelaxation; however, this required enzymatic degradation to AA to elicit activity [36]. Recent evidence suggests that eCBs, eCB-like, plant-derived, and synthetic cannabinoids might bind the orphan G protein receptors [37], and the peroxisome proliferator-activated receptors (PPARs) [38]. Overall these putative eCB receptors operate along with CBRs, through a variety of signalling mechanisms to exert pathophysiological effects in different tissues [39].

### 2.4. CBR Signalling

Both CB1 and CB2 belong to the rhodopsin subfamily of G protein coupled receptors that activate multiple molecular pathways regulating several cellular and systemic functions. CB1 and CB2 usually couple to intracellular PM associated G_i/o_ proteins, and inhibit adenylate cyclase through alpha subunits. Subsequent downstream signalling is cell-type dependent and can entail the following: release of nitric oxide (NO), activation of mitogen-activate protein kinases (MAPK), or initiation of protein kinases A and/or C, and cyclo-oxygenase-2 pathways [40]. Typically CBR activation inhibits voltage-gated calcium channels and stimulates inwardly rectifying K^+^ channels [40,41]. Depending on the cell type, activation of CB1 can stimulate phosphatidylinositol-4, 5-bisphosphate hydrolysis by phospholipase-C-beta, leading to release of inositol-1, 4, 5-phosphate, endoplasmic reticulum Ca^2+^ mobilization [42], and phosphoinositide-3-kinase (PI3K) cascade modulation [43]. Furthermore, so-called biased cannabinoids have the valuable characteristic of elegantly and selectively triggering particular CBR conformations that mediate CBR signalling beyond the binary on/off state [44]. Lastly, CBRs showed high levels of ligand-independent spontaneous activation, i.e., constitutive activity [45] and G_i/o_ sequestration [46]. These aspects of CBR pharmacology are of critical relevance for therapeutic targeting, and continued research will likely yield novel synthetic cannabinoids with more nuanced therapeutic activity and heightened clinical benefit.

### 2.5. Functions of the ECS

There is a kaleidoscope of physiological functions in which the ECS is known to play important roles, for instance, embryo implantation, cognition, and mechanisms regulating inflammation, nociception, ion homeostasis, energy balance, and cell differentiation and survival. Unsurprisingly, over the last years, the ECS has attracted considerable attention as a targetable signalling mechanism capable of ameliorating a plethora of pathomechanisms particularly relevant within the context of CVDs. At the sub-cellular level, emerging studies suggest that the ECS can regulate mitochondrial integrity and morphology, and modulate electron transport chain function, consequently impacting oxidative phosphorylation and energy production [47,48]. Indeed, eCB-induced mitochondrial dysfunction has been associated with increased reactive oxygen species (ROS) production, and substantial data now implicate ECS activation/inhibition in respectively conveying detrimental or beneficial effects upon mitochondrial biogenesis and respiratory chain activity [48]. Interestingly, studies demonstrated crosstalk between eCBs and redox-dependent processes. For instance, AEA and 2-AG modulate the activity of anti-oxidant enzymes by interacting with CB1 and/or CB2, as well as ion channels and PPARs. Indeed, the phyto-cannabinoids CBD and Δ^9^-THC display more anti-oxidant activity compared to the standard potent anti-oxidant butylated hydroxytoluene and the dietary anti-oxidants alpha-tocopherol and ascorbic acid, which was partially mediated by calcium channel inhibition [49]. Depending on the cell type and/or the involvement of tissue injury, CBRs show opposite effects in oxidative stress situations because CB1 activation results in enhanced redox imbalance, while CB2 stimulation lowered oxidative stress to convey beneficial free radical scavenging effects [50]. The ECS is implicated in the development of the growing number of diseases linked to redox homeostasis deregulation, including those associated with cardiovascular and metabolic disorders among others [50]. Studies in rodent models of cardiomyopathy have demonstrated that CB1-activated pathways promote oxidative damage, while CB2 stimulation limits oxidant-evoked myocardial injury. However, considering the unwanted side effects that can be associated with CB1 modulators, CB2 agonists have been suggested as more desirable for the management of acute cardiac tissue injury [51].

## 3. The ECS and CVDs

**Figure 2:**
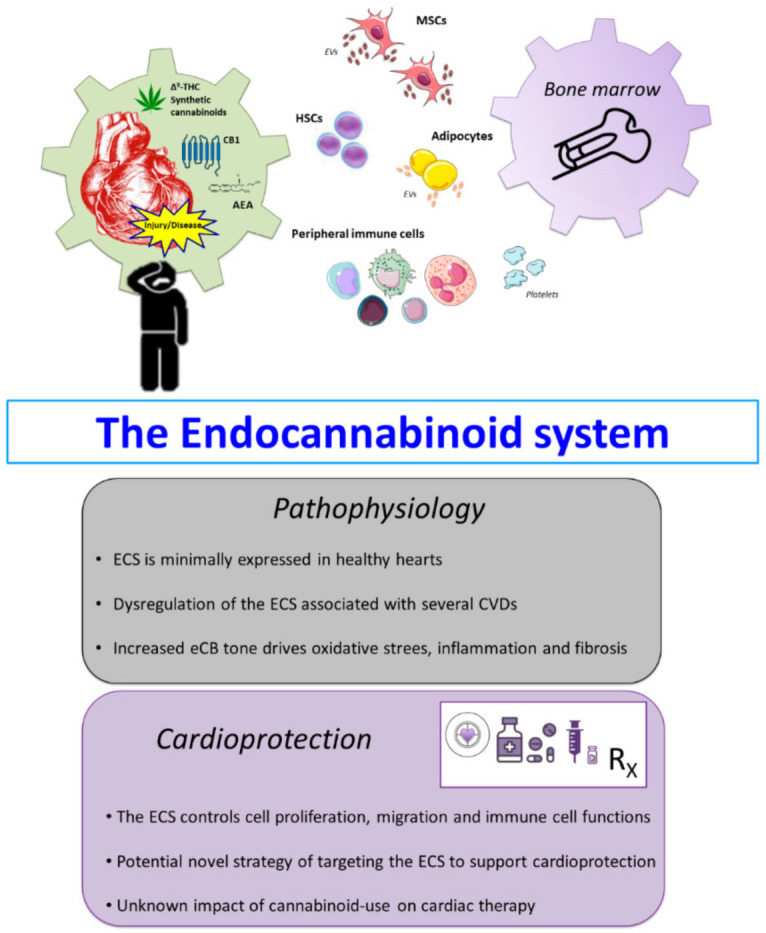
Contribution of the endocannabinoid system (ECS) to cardiovascular pathophysiology and cardioprotection. Schematic representation of the known interaction between the ECS and the bone marrow niche, immune cells, and other cells or cellular cargo that mediate pathophysiological mechanisms in CVDs, e.g., mesenchymal stem cells (MSCs), haematopoietic stem cells (HSCs), adipocytes, and extracellular vesicles (EVs). Comprehensive understanding of the dynamic alterations in the ECS during damage to the myocardium represents a crucial point to establish novel cannabinoid-based therapeutic approaches. For instance, during injury or pathological conditions the ECS becomes dysfunctional and loses specificity of activity that can further drive pathological events. Alterations including increased CB1 expression and anandamide (AEA) during these states activate various molecular signalling such as inflammation and fibrotic signalling. Thus targeting the ECS by inhibiting or enhancing relevant components, e.g., receptors, eCB metabolism etc., could be of potential benefit to improve current and future treatment strategies aimed at cardioprotection.

The ECS is minimally expressed in the healthy individual’s heart; however, this dramatically changes during the course of several CVDs. Following several or prolonged homeostatic deviations, the ECS becomes dysregulated and transitions to a pathological signalling mechanism [9] (Figure 2). This notable Janus aspect of the ECS is mediated by altering CBR expression and the concentration of eCBs. Indeed, while eCBs acting at CB2 receptors on immune cells induce anti-inflammatory effects, the same eCBs binding to upregulated CB1 on stressed cardiomyocytes activate pro-apoptotic signals, e.g., increased ROS and _i_[Ca^2+^]. Additionally, the use of both cannabis and potent synthetic cannabinoids have been linked to acute cardiac events, e.g., takotsubo cardiomyopathy and cardiac rhythm abnormalities. In the following sections we will give an overview of how the ECS can be modulated in several CVDs and the harmful effects of smoked cannabis or use of synthetic cannabinoids.

### 3.1. Myocardial Ischaemia/Reperfusion Syndrome

The ECS potentially modulates both acute and chronic cardiac disorders associated ischaemia/reperfusion (I/R) injury. For example, pre-conditioning with bacterial endotoxin lipopolysaccharide (LPS) achieved protective effects via increased eCB levels released from immune cells [52]. Additional studies investigating the role of CBRs in I/R injury demonstrated a major role for CB2 and a possible minor influence for CB1. Lépicier et al. showed that adding palmitoylethanolamide (PEA, an endogenous fatty acid amide) and 2-AG, but not AEA, to the re-perfusion medium reduced infarct size and myocardial damage [53]. CB1 and CB2 partially mediated this action, as a pre-treatment with a CB2 antagonist (SR144528) abrogated the cardioprotective effect of both PEA and 2-AG, whereas treatment with a CB1 antagonist (SR141716A) moderately inhibited the effect of 2-AG but not PEA. Similarly, Bouchard et al. demonstrated that following brief ischaemic pre-/post-conditioning (IPC), CB1 and CB2 antagonism blocked cardioprotection, implicating both receptors in IPC-induced cardioprotection [54]. Interestingly, in a rat model of I/R injury induced by coronary occlusion/re-occlusion, AEA reduced the infarct size and ventricular arrhythmias through CB2 activation rather than ATP-dependent K^+^-channels [55]. It has also been suggested that the anti-arrhythmic effect of CB2 stimulation involved inhibiting cAMP accumulation, a known driver of arrhythmia pathogenesis [56]. Similar results were reported in another in vivo study involving mouse myocardial I/R injury that showed a potent CB1 and CB2 agonist (WIN 55,212-2) reduced leukocyte-dependent myocardial damage, probably via CB2, since treatment with a CB2 antagonist (AM630) abolished this cardioprotection [57]. Lagneux and Lamontagne demonstrated that LPS-induced cardioprotection was mediated through CB2 but not CB1 [58]. Similarly, Joyeux et al. observed that eCBs mediated heat stress-induced pre-conditioning via CB2 rather than CB1 [59]. Furthermore, Duerr and colleagues explored the role of CB2 in murine cardiomyocytes and macrophages during the initial phase of ischaemic cardiomyopathy, i.e., prior to the onset of ventricular dysfunction or MI. Specifically, in cardiomyocytes they observed that the AEA-CB2 axis assisted the adaptation of contractile protein components and upregulated anti-oxidant mediators associated with apoptosis prevention. While in macrophages the AEA-CB2 axis augmented inflammatory cytokine production to instigate a more balanced inflammatory response. They thus provided novel evidence for specific cardioprotective mechanisms involving the eCB-CB2 axis in these two types of cardiovascular cells during the developmental phases of ischaemic cardiomyopathy [60]. Subsequently, Heinemann et al. simulated ischaemic conditions in vitro by exposing murine cardiomyocytes and macrophages to hypoxia and/or pro-inflammatory cytokine interferon gamma. They showed CB2-deficienct cardiomyocytes experiencing hypoxia were more vulnerable to apoptosis that was worsened by pro-inflammatory stimulation, further emphasizing the cardioprotective role of CB2 [61]. Although the role of the ECS in cardio-protection afforded by pre-conditioning is still not settled, the results obtained to date can be considered at least encouraging.

### 3.2. Atherosclerosis

Immune cells are the main source of eCBs in the cardiovascular system. Therefore it is not unexpected that the ECS can modulate immune functions and thus has a potential role in the treatment of CVDs such as atherosclerosis, which has a strong inflammatory component. CB2, being the main CBR linked to immune system regulation, is present at high levels in bone marrow (BM), lymphoid tissues, and immune cells [56]. Accordingly, it was suggested that protective immunomodulatory effects of eCBs could be mediated by CB2 while pro-atherosclerotic effects were mediated by CB1, a stance that fits with the observation that 2-AG and AEA were increased in obese patients with coronary circulatory dysfunction [62]. In support, Steffens et al. reported that in human and murine arterial atherosclerotic plaques, CB2 is present, whereas it was undetectable in non-diseased arteries [63]. Independent research groups showed oral Δ^9^-THC inhibited atherosclerotic plaque progression in apolipoprotein E knockout mice (ApoE^−/−^) fed a high-cholesterol diet [64]. Also, evidence that treating ApoE^−/−^ mice with a selective CB2 antagonist abolished the protective effect of Δ^9^-THC supported the hypothesis for the involvement of CB2 in anti-atherogenic processes [63,64]. Consistent reports indicating a selective CB2 agonist (JWH-015) modulates recruitment of human monocytes in a CB2-dependent manner, through the PI3K/Akt and extracellular signal-regulated protein kinases 1 and 2 (ERK1/2) pathways which further emphasized the anti-inflammatory properties of CB2 activation [65]. Additionally, selective CB2 activation reduced CD36-dependent accumulation of oxidized low-density lipoprotein (oxLDL) a major causal factor for atherosclerosis, and modulated the production of inflammatory cytokines by foam cells [66].

Although the role of CB1 in atherosclerosis is less clear, evidence showed CB1 activation in primary human coronary artery endothelial cells induced ROS production and cell death [8]. Interestingly, the most vulnerable plaques were particularly rich in CB1 [67] probably due to the considerable number of accumulating activated immune cells within active plaques as they densely express CB1. Thus, for cells such as endothelial cells, macrophages, and smooth muscle cells, CB1 antagonism could lead to plaque stabilization. Indeed, in smooth muscle cells, CB1 antagonism blocked the activation of platelet-derived growth factor-induced intracellular signalling, cell proliferation, and migration [67]. For endothelial cells and macrophages, CB1 blockade reduced MAPK activation and lowered ROS levels, benefiting both endothelial function and arterial vasodilatation [68]. To assess the potential protective effect of CB1 blockade, long-term treatment of mice lacking low-density lipoprotein (LDL) receptors (another murine model of atherosclerosis) with a CB1 antagonist/inverse agonist (SR141716, rimonabant), resulted in reduced atherosclerotic plaque formation, indicating that CB1 has an important role in plaque formation [69]. Dol-Gleizes et al. also investigated if SR141716 reduced inflammatory markers in peritoneal macrophages and demonstrated that SR141716 significantly reduced IL-6 and tumour necrosis factor (TNF) alpha but not monocyte chemoattractant protein-1 gene expression inducible by LPS and IL-1β treatment [69]. In an effort to speculate the mechanism of action, Dol-Gleizes and colleagues repeated the same experiment on peritoneal macrophages from CB1-deficient mice and obtained similar results in wild type (WT) mice, suggesting that this effect was not related to CB1 antagonism/inverse agonism [69].

SR141716 was the first CB1 antagonist/inverse agonist to reach the market for the treatment of obesity. Although weight loss itself is of potential interest in the atherosclerosis field, it was also shown that rimonabant improved other cardiovascular risk factors, e.g., LDL/high density lipoprotein (HDL) cholesterol ratio, tri-glycerides, and C-reactive protein (CRP). Building on the role of lipid profile in atherosclerosis, some studies demonstrated that CBD and Δ^9^-THC inhibit 15-lipoxygenases, an enzyme group responsible for oxLDL and 13-hydroxyoctadecadienoic acid (13-HODE) formation, both major causal factors connected to atherosclerosis and potentially other CVDs [64,70]. However, some unwanted central side effects elicited by Δ9-THC and SR141716, such as depressive disorders, mood alteration, and suicidal ideation and completion, indicate that further studies are required to confirm the benefits of modulating the ECS for atherosclerosis. Promisingly, the development of effective peripherally restricted CB1 modulators, e.g., water-soluble CB1 antagonists, stands to improve the likelihood of successful treatment free from unwanted side effects.

### 3.3. Hypertension

The ECS may play a role on blood pressure regulation through both central and peripheral mechanisms. Central administration of cannabinoids can cause either sympathoinhibition or sympathoexcitation depending on the site of microinjection, showing sympathoinhibition when injected into the *nucleus tractus solitarii* of rats, and sympathoexcitation when injected into the midbrain periaqueductal gray and also the rostral ventrolateral medulla of rats. Referring to the peripheral mechanisms, cardiac CB1 activation exerts negative chronotropic and ionotropic effects, which are potentially independent of the central nervous system [71]. Both mechanisms concur to generate a triphasic response following intravenous administration of cannabinoids that consists of an immediate fall in blood pressure attributable to TRPV1 (Phase 1), a brief pressor effect (Phase 2), and finally a prolonged hypotensive effect mediated by peripherally located CB1R (Phase 3) [71].

During hypertension (HT) the ECS becomes over-activated via compensatory mechanisms intended to limit increased blood pressure (BP) and myocardial workload. In particular, tonic activation of the ECS, owing to upregulated CB1 expression in the cardiac and vascular endothelia, was reported in various types of HT. It is also well known that smoking cannabis causes acute increases in HR. However, this is not accompanied by long-lasting changes in BP, except in cases of chronic use, which is associated with decreased BP [72]. Gorelick et al. confirmed this and reported that symptomatic hypotension in cannabis smokers was reversed by administering an antagonist of CB1 (rimonabant) [73]. Interestingly, Δ^9^-THC and AEA induced a larger and more sustained hypotensive response in spontaneously hypertensive rats compared to normotensive controls [74], suggesting that the ECS becomes involved in cardiovascular regulation during the developmental stages of HT. However, up-regulated CB1 expression in cardiovascular tissues likely contributed to higher Δ^9^-THC and AEA sensitivity [75]. The normal BP in CB1^−/−^ mice and unaltered BP in WT mice after administering AEA or CB1 modulators suggested that the ECS is not involved in cardiovascular regulation under normal conditions [76,77]. Furthermore, mice lacking FAAH, which have increased ECS tone, e.g., increased serum eCB concentrations, have a strong hypotensive and cardio depressive phenotype [78]. An unidentified receptor was suggested to mediate this hypotension since similar results were observed in CBR knockout mice, possibly involving ion channels, e.g., Ca^2+^, K^+^, Ca^2+^-activated K^+^, and TRPV1 [79]. Lastly, phyto-cannabinoids such as Δ^9^-THC produced vasodilation followed by hypotension, again CBR independent, potentially via TRP ankyrin type 1 [80].

Drugs that increase basal levels of eCBs are frequently investigated as HT treatments. A common target is the eCB metabolic enzyme FAAH, which when inhibited causes an overall increased ECS tone. In 2016 the FAAH inhibiting compound BIA 10-2474 was administered to healthy volunteers as part of a phase I clinical trial for potential treatment of many disease including HT. However, the trial was stopped following a disastrous event in which one participant was killed and another seriously harmed. Subsequent activity-based proteomic studies revealed BIA 10-2474 inhibits several lipases and subsequently produces substantial alterations in lipid networks, suggesting that promiscuous lipase inhibitors have the potential to cause fatal metabolic dysregulation particularly in the brain [81,82].

Barriers such as the rapid development of tolerance to Δ^9^-THC, lack of highly specific inhibitors, and difficulty separating centrally and peripherally mediated effects lessen the ability to safely target this complex signalling system for BP management. Despite this, a new hope comes from the discovery of small molecules with different mechanisms of modulating the ECS, e.g., pepcans, which are a large family of eCB peptides capable of CBR antagonism or allosteric modulation of CBRs depending on their amino acid sequence length [83,84,85].

### 3.4. Cardiomyopathy and Heart Failure

The main common consequence of either cardiomyopathy or heart failure (HF) is a decreased ability of the myocardium to pump blood. Although both cardiomyopathy or HF can be acute or chronic in nature, inheritance and progression to HF are strongly associated with cardiomyopathy. In these contexts the ECS is involved in different pathomechanisms of the resultant cardiac pathology.

#### 3.4.1. Diabetic Cardiomyopathy

The mechanisms of diabetic cardiac dysfunction are complex and involve increased oxidative/nitrative stress, activation of downstream transcription factors responsible for pro-inflammatory cytokine expression, accumulation of advanced glycation end products, changes in extracellular matrix composition, and inactivation of pro-survival signalling or activation of cell-death pathways, which are now know to be additional to ECS dysregulation [86,87,88]. In the cardiovascular system, activation of CB1 by eCBs may directly or indirectly (via their metabolites) enhance diabetes-associated inflammation and ROS generation that promote tissue injury and the development of diabetic complications. In humans, links between the ECS and diabetic cardiomyopathy are evident from reports detailing increased ECS tone in diabetic patients’ serum and adipose tissue [89,90].

It is now known that various components of the *Cannabis sativa* plant, e.g., the most characterized active ingredients Δ^9^-THC or CBD, exert potent anti-inflammatory and anti-oxidant effects. Indeed, Rajesh et al. showed the protective effects of CBD in a streptozotocin-induced diabetic rat model of type 1 diabetic cardiomyopathy and in primary human cardiomyocytes exposed to high glucose in vitro. Chronic CBD treatment attenuated markers of diabetic cardiomyopathy such as oxidative and nitrative stress, inflammation, upregulated cell death and interrelated signalling pathways, and cardiac fibrosis [87]. Pharmacological inhibition or genetic deletion of CB1 also attenuated diabetes-induced cardiac dysfunction and pathological alterations [91]. Additionally, CB2 activation may exert beneficial effects against various diabetic complications by attenuating high glucose-induced endothelial cell activation and inflammatory response and inflammatory cell chemotaxis, transmigration, and adhesion [92]. Similar to other CVDs, diabetes-associated cardiomyopathy is strongly connected to an inflammatory component that could represent a key target for ECS modulators. However, this will heavily depend on identification of the specific CBR axis responsible for the effects mentioned above, and subsequently on the development of synthetic molecules capable of site-specific action and downstream receptor activity, e.g., peripherally restricted compounds and activation of delineated receptor signalling with biased agonists.

#### 3.4.2. Chemotherapy-Linked Cardiomyopathy

Dose-dependent cardiotoxicity and major adverse cardiac outcomes associated with quinone-containing anthracyclines (e.g., epirubicin, doxorubicin, daunorubicin, and idarubicin) and other potent anti-tumour drugs (e.g., alkylating agents, endocrine therapy, and cyclin inhibitors), which can occur several years or decades after treatment, represent a major concern [93]. In relation to a potential protective function of the ECS, Mukhopadhyay et al. discovered doxorubicin elevated eCB levels in cardiomyocytes in vivo and in vitro, and that pharmacological inhibition and genetic ablation of CB1 attenuated doxorubicin-induced myocardial dysfunction, mitochondrial deregulation, oxidative/nitrative stress, and associated cell death pathways. Crucially, these positive molecular outcomes also correlated with improved cardiac contractile functions and markers of myocardial fibrosis [94]. Hao and colleagues determined that CBD exerted its protective effects against doxorubicin-mediated cardiotoxicity and cardiac dysfunction by attenuating oxidative/nitrative stress, improving mitochondrial function, enhancing mitochondrial biogenesis, and decreasing inflammation, matrix metallopeptidases, and cell death [95]. However, the direct mechanism of action for CBD-mediated cardioprotection against doxorubicin was not tested.

Interestingly, for some forms of cancer, a combination of CBD and Δ^9^-THC acted synergistically with chemotherapeutic agents and limited tumour cell proliferation, induced tumour-selective cell death, and reduced chemo-resistance, indicating that the combination of ECS modulators with chemotherapeutics may be beneficial in terms of anti-tumour effect and the possibility to reduce chemotherapeutic doses [96]. This combination could translate into a reduction of the cardiotoxic effects exerted by anticancer agents. Regarding, Fouad et al. showed that CBD represents a potential protective agent against doxorubicin cardiac injury, since CBD treatment (5 mg kg^−1^/day, i.p.) significantly reduced the elevations of serum creatine kinase-MB and troponin T and cardiac malondialdehyde, TNF alpha, and NO and calcium ion levels, and attenuated the decreases in cardiac reduced glutathione, selenium, and zinc ions [97]. Accordingly, CBD prevented doxorubicin-induced cardiomyopathy/heart failure by attenuating oxidative/nitrative stress, mitochondrial dysfunction, cell death, and inflammation, and by promoting mitochondrial biogenesis, in an in vivo model of doxorubicin-induced cardiomyopathy [95]. Lastly, the excellent safety profile of CBD and Δ^9^-THC, their anti-emetic, anti-inflammatory, and analgesic effects heighten the appeal of these phyto-cannabinoids and the necessity for in-patient clinical trials of phyto-cannabinoids for acute or delayed chemotherapy-induced cardiomyopathy.

#### 3.4.3. Hepatic Cirrhosis Cardiomyopathy

A significant upregulation of the ECS is observed in patients with hepatic cirrhosis [98], and it is well documented that eCBs or synthetic cannabinoid ligands exert opposing effects on multiple pathological processes in the liver and heart. In particular, CB1 activation promotes hepatic steatosis, inflammation, fibrosis, and also contributes to peripheral vasodilation and the ensuing cirrhotic cardiomyopathy. Indeed, it was shown that CB1 blockade increased arterial pressure and vascular resistance in rats with carbon tetrachloride-induced cirrhosis, suggesting CB1 contributes to the pressor effect of eCBs acting on CB1 to reduce ECS tone and portal circulation, the latter being an important clinical goal in late-stage liver cirrhosis [99]. On the contrary, CB2 activation positively regulates interactions between activated endothelium and infiltrating immune cells that reduce the extent of liver injury, inflammation and fibrosis in animal models of liver cirrhosis [98]. Cardiac expression of CB1 is apparently not affected by liver cirrhosis, leading to the hypothesis of reduced cardiac contractility initiated by increased release of eCBs, e.g., AEA, and the suggestion that inflammation represents the major trigger for AEA synthesis [100]. The results of in vitro studies indicate AEA concentrations in the hearts of humans and animals with hepatic cirrhosis are increased, which causes CB1 activation, myocardial contractility impairment, and fibrosis. In contrast, endogenous CB2 activation alleviates pro-fibrotic signalling and positively regulates liver inflammation [86]. Studies showed that blocking MAGL, thereby increasing 2-AG concentrations, protects against inflammation and damage from hepatic I/R via eCB signalling. Thus, CB2 agonists and MAGL inhibitors were proposed as future treatments for cirrhotic cardiomyopathy [99]. To investigate the ECS in cirrhotic cardiomyopathy, Gaskari et al. used the bile duct ligation (BDL) murine model, which shows disease markers in left ventricular papillary muscles resembling the clinical syndrome seen in patients, e.g., reduced cardiac contractile response to beta-adrenergic stimulation [101]. Incubation of papillary muscle preparations from BDL mice with a CB1 antagonist (AM251) restored beta-adrenergic responsiveness. In a follow-up study, this group found increased monocyte recruitment and elevated eCBs levels in BDL mice hearts and that CB1 blockade also prevented monocyte infiltration, local release of eCBs from macrophages, and restored impaired responses to haemorrhage [102]. In 2020, Matyas et al. demonstrated that a selective CB2 agonist (HU910) prevented cardiac and renal tissue damage in BDL mice, indicating that CB2 has a pivotal pathophysiological role in hepatic cardiomyopathy, and CB2-specific agonists may delay or prevent the development of cardiomyopathy or renopathy associated with severe liver disease [100]. ECS modulators such as peripherally restricted CB1 antagonists or CB2 agonists could be suitable treatments to extend the lives of patients awaiting liver transplants. Thus, further clinical trials and development of drugs that specifically target peripheral CBRs are warranted.

#### 3.4.4. Septic Shock Cardiomyopathy and Myocarditis

The classical eCBs, i.e., AEA and 2-AG, are generated by platelets and macrophages during septic-, haemorrhagic-, and shock-induced hypotension through activation of CB1 [103]. Specifically, AEA is considered to be a strong mediator of endotoxin-induced hypotension via activation of vascular CB1 [104] and is increased in the blood of endotoxic shock patients [105]. Also, preventing endotoxin-mediated upregulation of plasma AEA or 2-AG, by administering a CB1 inverse agonist (AM281), was beneficial in experimental animals, as it improved mortality associated with septic shock [106]. Furthermore, Varga and colleagues reported pre-treatment of animals with a CB1 antagonist (SR141716) not only prevented LPS-induced hypotension, but also improved survival [107]. For instance, endothelial lesions and/or dysfunctions were a common denominator in various studies of complications in septic patients. Indeed, Yamaji and co-workers demonstrated that AEA-induced apoptosis in a time- and dose-dependent manner in human umbilical vein endothelial cells (HUVEC) involving extracellular delivery via an AEA membrane transporter, binding to TRPV1, phosphorylation of c-Jun NH2-terminal kinase, and p38 mitogen activated protein 1-beta-kinase (but not ERK) induced caspase-3 activity and mitochondrial dysfunction [108]. In contrast to AEA-induced cell death in other cardiovascular cell types, e.g., cardiomyocytes, Yamaji and co-workers found CB1 inhibition enhanced AEA-mediated HUVEC cell-death, indicating that CB1 activation in endothelial cells is important for cell survival. Lee and colleagues showed that chronic CBD treatment diminished CD3^+^ and CD4^+^ T-cell activation and inflammatory response, cardiomyocyte cell-death, fibrosis, and myocardial dysfunction in a well-known mouse model of experimental autoimmune myocarditis [109]. Recently, synthetic and phyto-cannabinoids were proposed to have therapeutic potential to counteract cardiovascular cytokine storm associated with SARS-CoV-2 infection [110,111,112] owing to the strong involvement of the ECS in immuno-suppression, e.g., dampening cytokine release, decreasing immune cell proliferation and activity, and more direct impacts on viral pathogenesis, i.e., viral entry and replication, and host cell destruction [113].

Undoubtedly more basic research and trials in this area are necessary; however, the idea of a close involvement of the ECS in septic shock-induced haemodynamic changes is appealing and deserves further investigation since there are no specific therapeutics.

#### 3.4.5. Stress Cardiomyopathy

A commonly reported acute cardiovascular complication of cannabis use is stress cardiomyopathy (SC), also known as takotsubo cardiomyopathy [114], which is increasingly reported following use of substances containing highly potent synthetic cannabinoids. A case report from 2014 described an unusual incidence of the mid-ventricular variant of takotsubo cardiomyopathy associated with cannabinoid hyperemesis syndrome in a long-term cannabis user [115]. Until this report, a true pathophysiologic relationship was uncertain although the connection was to some extent acknowledged in the basic research literature. For instance, Singh and co-workers suggested a possible association between cannabis and synthetic cannabinoid use with SC, since CB1 activation has mixed inotropic effects on the myocardium and CB1 activation induces catecholamine surges that may precipitate SC [116,117]. An abstract presented at the American Heart Association Scientific Sessions and Resuscitation Science Symposium by the same group presented a case report of transient left ventricular regional ballooning in a young male cannabis user despite favourable cardiac risk factor profile (youth and low co-morbidity prevalence, e.g., HT and diabetes) [118]. Furthermore, a selective CB2 agonist demonstrated concentration-dependent decreases in cardiac contractility in a rabbit animal model of SC [119]. Evidence for a role of the ECS in SC pathogenesis has been mounting which necessitates further investigative studies. Indeed, a recent study by Desai and colleagues [120,121] quantified SC prevalence and the in-hospital outcomes of hospitalized cannabis users versus non-users and concluded that although the cannabis-use group had an overall lower prevalence of SC compared to non-users there was a 50% higher risk of in-hospital mortality in the cannabis-use group. They also reported higher rates of cardiogenic shock, stroke, and haemodynamic support in the cannabis-use group. Clearly evidence linking excessive CB1 activation to SC is building, which necessitates raising the awareness of this link within cannabis users and the healthcare community.

### 3.5. Arrhythmias

Following the relaxation of the legal stance of cannabis, hospitalizations of cannabis users due to arrhythmias increased 2-fold with an all-cause in-hospital mortality of 0.5%, although estimates approaching 5% were also reported [122]. Indeed, a number of cardiac rhythm abnormalities connected to cannabis use have been documented: atrial fibrillation (AF)/flutter, atrioventricular block/asystole, sick sinus syndrome, ventricular tachycardia, and Brugada pattern [117,119]. Specifically, smoking cannabis can lead to arrhythmias associated with AF [123]. For this reason, some investigators have called for cannabis to be included on the list of possible triggers in young adults presenting with paroxysmal AF, once cardiac disease and other common causes of AF have been excluded [124]. The most suitable treatment plan for such cases, and the long-term effects of cannabinoids on the cardiovascular system and AF recurrence remains unknown [123,125]. Moreover, another study reported the first case of ventricular fibrillation due to unintentional cannabis overdose that was recorded by an implantable cardioverter defibrillator in a 60-year-old male [126]. Desai et al. reported that 2.7% of hospitalized recreational cannabis users experienced arrhythmias with AF (1.9%) being the most common [122]. It has been postulated that increased catecholamines and beta-adrenergic stimulation following cannabis use may theoretically increase arrhythmogenicity [116]. Dose appears to play a part, with low to moderate doses activating sympathetic cardiac innervation causing tachycardia, while higher doses trigger parasympathetic signals inducing bradycardia and hypotension [127]. These findings suggest that, although the cardiovascular effects of cannabis are usually well tolerated in young healthy people, cannabis or synthetic cannabinoid use may trigger life-threatening arrhythmias in individuals with pre-existing or undiagnosed cardiac pathology or predisposing risk factors [126].

In terms of the therapeutic potential of targeting the ECS, the effects of CBD on the onset and evolution of cardiac arrhythmias were investigated during/after ischaemia. Walsh et al. [128] demonstrated that CBD dose-dependently reduced both the total number of ischaemia-induced arrhythmias and infarct size when administered immediately prior to ischaemia onset. Furthermore, they also showed that CBD reduced infarct size when given at the time of reperfusion, a clinically relevant aspect of CBD action [128]. Interestingly, Krylatov et al. demonstrated in a rat model of coronary occlusion/reperfusion that both AEA and a non-selective CBR agonist (HU210) decreased the incidence of ventricular arrhythmias and reduced infarct size through CB2 activation [129].

Recent studies showed little or no specific mention of cannabis in CVD risk assessments or lifestyle advice guidelines [130]. Considering the evidence linking cannabinoids to cardiovascular dysfunction, recommendations regarding smoked or vaped cannabis and consuming other cannabinoid-containing products should become part of CVD guidelines [125]. Also, understanding dose-response effects and the long-term implications that regular to chronic use of cannabis or synthetic cannabinoids has on the cardiovascular system is of rising importance. Growing awareness among cardiologists of the distinct risks associated with the use of cannabinoid-containing substances will undoubtedly shed more light on this emerging topic, which can already be appreciated from increased rate of case reports of cannabinoid-induced cardiovascular events [131,132,133,134,135] and the debate it has stimulated [136,137,138].

## 4. Clinical Trials

Various ECS modulators entered clinical trials for diseases associated with: metabolic dysfunction, uncontrolled cell-fate, oxidative stress and inflammation. Here we will specifically concentrate on clinical trials assessing ECS modulators and cardiovascular-related endpoints (see Table 1).

Since cannabis is by far the most commonly used recreational drug, it is not surprising that the pharmacokinetics and physiological effects of cannabis were explored in a clinical trial (NCT00225407) [139]. Although this trial focused mainly on the central effects of cannabis it also confirmed the long-known effect of cannabis on HR which correlated to increasing Δ^9^-THC concentrations; +57 beats per minute (bpm) over baseline HR readings when cigarettes with 69 mg Δ^9^-THC were smoked (safety protocols limited the maximum HR to 170 bpm and a minimum mean arterial BP of 55 mmHg). Isolated cannabis components, e.g., CBD, have also entered clinical trials. The first study (NCT01844687) assessed the effects of different CBD concentrations on the subjective, reinforcing, cognitive, and cardiovascular effects of smoked cannabis. CBD did not alter the subjective, reinforcing, or cardiovascular effects of smoked cannabis relative to placebo [140]. Other clinical trials specifically focused on investigating the effect of CBD on BP and, vascular and cognitive functions in healthy volunteers (E18102012). Acute oral CBD reduced resting BP and prevented stress-induced BP increases [141]. A randomized phase I study (NCT03295903) demonstrated that a patented capsule formulation of CBD increased cerebral perfusion and slightly reduced BP compared to baseline, in healthy young adults and an older group [142]. Taken together, these data indicate that phyto-cannabinoids exert a role on various cardiovascular measures. Data from two recently started trials will be of great interest in order to confirm previous findings. The first trial (NCT03508895) investigated the effect of consuming whole hemp seed protein alone or combined with bioactive peptides also derived from hemp seed protein on systolic and diastolic ambulatory BP [143]. The second trial (NCT03379857) explored the prevalence of strokes secondary to reversible cerebral vasoconstriction attributable to cannabis consumption. The results of this trial are eagerly awaited, as there is keen interest to clarify the risks associated with cannabis and stroke risk in the young population that has a rising incidence and unknown causation [144,145,146]. Other phyto-cannabinoids present in cannabis at lower levels compared to CBD and Δ^9^-THC, e.g., tetrahydrocannabivarin (THCV) and terpenoids, have shown therapeutic promise. Notably, THCV counteracted robust Δ^9^-THC-induced tachycardia, indicating that THCV has potential to mitigate some negative side effects of Δ^9^-THC [147].

Turning our attention to obesity and metabolic dysfunction, AEA and 2-AG levels correlated with cardiac circulatory and endothelial dysfunction and were purported serum biomarkers of cardiovascular risk in obese individuals [148,149]. Additionally, variation in the genes coding for CB1 and FAAH were linked with physical and serum measures of obesity, e.g., increased waist circumference and dyslipidaemia [150,151,152,153,154,155,156]. Interestingly, CB2 gene variation was not associated with similar cardiovascular risk factors or MI [157]. These genetic studies provided more evidence for the pursuit of clinical trials investigating CBR modulators as a treatment for obesity and associated cardiometabolic disease. Thus, several clinical trials monitored the effects of compounds with affinity for CBRs on obesity, metabolic dysfunction, and atherosclerosis.

An early clinical trial (NCT00636766), which originated from findings in mouse models [104], examined the influence of rimonabant on atherosclerotic plaque stability and found the CB1 antagonist rimonabant promoted plaque regression and stability. Continuing this promising start, the 12-month long rimonabant in obesity (RIO) programs (Europe, Lipids, North America and Diabetes; NCT00386061) reported that rimonabant improved anthropometric data and modifiable cardiometabolic risk markers [158]. However, telling negative neuropsychological events were listed among other adverse events associated with rimonabant. Nevertheless, further trials of rimonabant continued. The STRADIVARIUS study (NCT00124332) failed to show an effect for rimonabant on the primary outcome (change in atheroma volume), it did find a favourable effect on normalized total atheroma volume (the secondary outcome) [159]. The ADAGIO-Lipids trial (NCT00239967) assessed the net impact of rimonabant on cardiometabolic risk factors and, abdominal and liver fat, which corroborated previous positive findings for cardiometabolic risk factors from the RIO programs and STRADIVARIUS trial [160] [161]. The AUDITOR study (NCT00228176) also found no differences in atherosclerosis progression [162]. Lastly, the CRESCENDO study (NCT00263042) aimed to verify whether rimonabant reduced the risk of MI, stroke, or death [163]; however, it was prematurely terminated in late 2008 ahead of rimonabant being withdrawn from the European market in January 2009. Further analyses of data from these overlapping trials were undertaken in order to better identify patients likely to benefit most or separate patient subgroups (e.g., statin naïveté, presence diabetes). However, *post hoc* analyses failed to influence the debate on the risks/benefits of continued rimonabant development. Thus, ultimately all rimonabant trials were terminated leaving open the question of why metabolic variables and tissue lipid metabolism improvements did not translate into clinical benefits.

Renewed optimism regarding the ECS as a drug target for CVDs comes from newer clinical trials, which have not yet disclosed data. For example, the phase I, single centre, open-label 2021 trial investigating the safety and efficacy of CBD in patients with heart failure (CAPITAL-AC, NCT03634189) and the double-blind, placebo-controlled 2021 trial designed to assess the efficacy and safety of a pharmaceutically produced oral CBD formulation in patients with COVID-19 and CVDs (NCT04615949). Indeed further pre-clinical and clinical research directed at establishing whether the ECS is indeed “a neglected pharmacological treasure trove” [164] should help identify other ECS-linked mechanisms with therapeutic applications or discover new pro-drugs from which semi-synthetic or synthetic cannabinoid-based medicines could be manufactured.

## 5. Conclusions and Outlook

We reported studies investigating the involvement of the ECS in cardiovascular pathophysiology. Although, lamentably research output focused on the ECS and CVDs persistently trails research focused on the ECS and diseases of the central nervous system as evident from our PubMed search of the literature from 1994 to 2020 for “endocannabinoid system, cardiovascular system” or “endocannabinoid system, central nervous system” which returned peak yearly publication rates of 49 (in 2007) versus 226 (in 2015) respectively. To some degree this can be explained since the main effects of cannabis is on cognition and that eCBs and CBRs were first isolated from the brain. However, research scrutinizing the ECS and the pathomechanisms of CVDs should be expanded because many questions still need to be answered and there is acute need for novel cardiovascular drugs. For example, it would be a rewarding undertaking to concentrate efforts on determining the role that each ECS player exerts in CVDs and identifying the exact mechanisms of action in different CVDs. Such functional itemization is essential to take full advantage of modulating the ECS and transfer research findings into clinical practice. In this scenario, it will also be of crucial interest to precisely evaluate the side effects that accompany ECS manipulation and, of course, pinpoint the interactions that occur with other drugs or endogenous factors. In a broader vision, since current cardiac therapy spans a wide spectrum, from gold-standard-of-care to state-of-the-art cell-based treatments, it could be also useful to study if there are any significant consequences when either the donor or recipient of stem cells or their derived cells, tissues or organs uses substances containing cannabinoids.

When assessing the risks/benefits of targeting the ECS, the broader social and public health consequences of allowing access to cannabis and related products for medical use should be fully accounted. Several studies, mainly from the USA, have focused on the effect that changing cannabis regulation had on: recreational use by young people, motor vehicle fatalities, suicides, use of other substances and health care contacts [165,166]. Overall these studies did not find significant deviations from the norms of states in the USA that did not change cannabis laws [165]. In accordance, the European Monitoring Centre for Drugs and Drug Addiction assessed existing systematic reviews (largely the same studies coming from the USA) to reach similar conclusions [166]. Lastly, further positive indications that targeting the ECS and prescribing cannabis-based medicines warrant further investigation is evident from the recommendations of the WHO expert committee on drug dependence that lead the UN commission on narcotic drugs to re-schedule laws concerning cannabis and cannabis-containing derivatives.

Demonstrably there is ample space for further growth and knowledge-attainment in this rapidly changing area. The continued awareness among the stakeholders will ensure confidently informed clinicians, patients, and consumers of cannabinoid containing substances.

## Figures and Tables

**Table 1:** Clinical trials involving ECS modulators and CVDs or cardiovascular risk factors.

Trial	ECS Modulator	Readouts	Findings	Phase
“A Double-Blind, Randomised, Placebo-Controlled, Cross-Over Study on the Pharmacokinetics and Effects of Cannabis”-NCT00225407, 2005.	Smoking cannabis cigarettes (different Δ^9^-THC strengths).	Primary: Serum Δ^9^-THC concentration over time; physical parameters (HR, BP); psychomotor tests (e.g., continuous attention); event related potentials. Secondary: Self-reporting questionnaires.	HR and intoxication were positively linearly associated with increasing Δ^9^-THC doses (29.3, 49.1, 69.4 mg).	Phase 1.
“Laboratory Study of the Influence of Oral Cannabidiol on the Subjective, Reinforcing and Cardiovascular Effects of Smoked Marijuana”-NCT01844687, 2013.	Active cannabis with 0, 200, 400 or 800 mg CBD; inactive cannabis (containing 0.56% Δ^9^-THC) with 0, 200, 400 or 800 mg CBD.	Primary: “Feeling high”mood scale-subscale. Secondary: rating form assessing, experience, strength, additional puffs taken, HR, plasma concentration of CBD.	Oral CBD pre-treatment does not alter the subjective, reinforcing, or cardiovascular effects of smoked cannabis. Active cannabis produced significant increases in ratings of “High” and “Good Effect” as well as assessments of the cannabis cigarette (e.g., “Strength”, “Liking”, “Desire to take again”) and HR.	Phase 2.
“A single dose of cannabidiol reduces BP in healthy volunteers in a randomized crossover study”. University of Nottingham (UK) study reference code: E18102012, *c.*2017.	CBD (600 mg).	Cardiovascular parameters: Systolic, diastolic and mean arterial BP, HR, stroke volume, CO, ejection time, total peripheral resistance, and forearm blood flow.	Acute administration of CBD reduces resting BP.	Phase 1.
“The Effects of Cannabinoids on Vascular and Cognitive Function in Young and Old Healthy Adults”-NCT03295903, 2018.	CBD and TurboCBD™.	Primary: Circulating CBD and nitric oxide markers and vascular function. Secondary: Height, weight, body mass index, systolic and diastolic BP, HR, respiration, questionnaires (medical history, gastrointestinal distress, anxiety), cognitive and exercise performance evaluations.	TurboCBD™ had higher bioavailability than CBD and at 90 mg was associated with increased cerebral perfusion and slight reduction in BP.	Phase 1.
“Hemp Seed Protein Consumption for Hypertension“-NCT03508895, 2018.	Hemp seed protein.	Primary: Change in 24 h ambulatory BP. Secondary: Change in BP, pulse wave velocity, augmentation index, body weight, waist circumference, hip circumference, body composition, total serum cholesterol, HDL/LDL cholesterol, serum triglycerides, serum glucose, serum creatinine, plasma insulin concentrations, insulin homeostasis modelling assessment, renal panel.	No data to date.	Phase 2.
CANNASTROKE “Prevalence of Strokes Secondary to a Reversible Cerebral Vasoconstriction Attributable to Cannabis Consumption in Young Subjects (≤45 Years) Hospitalized for an Ischaemic Stroke”-NCT03379857, 2017.	Cannabis.	Primary: Evaluation of cannabis use, reversible vasoconstriction on medical imaging of intracranial arteries.	No data to date. Estimated primary completion date: January 2025.	Not applicable (behavioural study).
“Atherosclerotic Plaque Texture-Experimental and Clinical Study on the Diagnostic and Therapeutic Strategies of Atherosclerotic Plaque Vulnerability”-NCT00636766, 2005.	Rimonabant combined with exercise.	Primary: Ultrasound and immuno-histochemical parameters of plaque stability and novel cardiovascular risk factors. Secondary: Long-term cardiovascular outcomes.	Rimonabant and exercise induced plaque regression and promoted plaque stability. A combination of the two interventions failed to show additive or synergistic benefits.	Phase 3.
RIO-Europe “A Randomized, Double-Blind, Placebo-Controlled, Parallel-Group, Fixed-Dose, Multicenter Study of Weight-Reducing Effect and Safety of SR141716 in Obese Patients With or Without Comorbidities”-NCT00386061, 2001.	Rimonabant (5/20 mg daily), reduced caloric intake and exercise promotion.	Primary: Change in body weight, waist circumference, and BP from baseline to 1 year. Secondary: Lipid profile, HDL cholesterol and triglycerides; patients (%) with improvement of glucose tolerance, patients (%) with NCEP-ATPIII metabolic syndrome.	Rimonabant produced weight loss and significant improvements in multiple cardiometabolic risk factors.	Phase 3.
STRADIVARIUS “Strategy To Reduce Atherosclerosis Development InVolving Administration of Rimonabant-the Intravascular Ultrasound Study”-NCT00124332, 2005.	Rimonabant (20 mg, daily for 18–20 months).	Primary: Change from baseline in percent atheroma volume. Secondary: Change from baseline in normalized TAV.	After 18 months of treatment, no effect of rimonabant on the primary efficacy parameter. A statistically significant favourable effect on the secondary end point was observed.	Phase 3.
ADAGIO-Lipids Trial “Effect of Rimonabant on the High-Triglyceride/ Low–HDL-Cholesterol Dyslipidemia, Intra-abdominal Adiposity, and Liver Fat”-NCT00239967, 2005.	Rimonabant (20 mg daily for 12 months).	Measurements of LDL particle size, HDL quantity, quality and subfractions, and apo B/apo A1 ratio; assessments of visceral and liver fat (by a computed tomography sub-study).	Rimonabant significantly improved multiple cardiometabolic risk markers and induced significant reductions in both intra-abdominal and liver fat.	Phase 3.
AUDITOR “Atherosclerosis Underlying Development Assessed by Intima-Media Thickness in Patients on Rimonabant”-NCT00228176, 2005.	Rimonabant (20 mg daily for 30 months).	Primary: Absolute change from baseline in averaged per patient CIMT. Secondary: First occurrence of any component of stroke/MI/cardiovascular death. First occurrence of any component of stroke/MI/cardiovascular death/hospitalization for revascularization procedure, unstable angina, transient ischaemic attack.	No difference in atherosclerosis progression between patients receiving rimonabant for 30 months and those receiving placebo for the primary efficacy measure (absolute change in CIMT).	Phase 3.
CRESCENDO “Comprehensive Rimonabant Evaluation Study of Cardiovascular ENDpoints and Outcomes”-NCT00263042, 2005.	Rimonabant (20 mg daily up to 13.4 months).	Primary: First occurrence of any of myocardial infarction, stroke or cardiovascular death. Secondary: First occurrence of any of myocardial infarction, stroke, cardiovascular death, cardiovascular hospitalization and all-cause mortality.	No evidence for the efficacy of prevention of adverse cardiovascular outcomes by rimonabant. Rimonabant was associated with serious side-effects (e.g., neuropsychiatric, gastrointestinal) and the trial was discontinued.	Phase 3.
PRIMARIA “Early Detection of Atherosclerosis in the Primary Care Setting: a Randomized Trial to Assess the Efficacy of a Novel Strategy in the Primary Prevention of Cardiovascular Diseases”-NCT00734123, 2008.	Rimonabant.	Primary: CIMT progression/regression. Secondary: Cardiac and cerebrovascular events.	No data to date.	Phase 4.
CAPITAL-AC “Cannabidiol in Patients With Heart Failure in AHA/ACC Stages A-C”-NCT03634189, 2021.	CBD.	Primary: Number of participants with treatment-related serious adverse events and events of interest as assessed by MedDRA v5.1.	No data to date. Estimated study completion date: December 2021.	Phase 1.
“Study to Evaluate the Efficacy and Safety of CardiolRx™ in Patients With COVID-19 and Cardiovascular Disease or Risk Factors A Double-blind, Placebo-controlled Trial”-NCT04615949, 2021.	CBD, pharmaceutically produced with <5 ppm THC.	Primary: All-cause mortality, ICU admission, ventilator support, cardiovascular complications.	No data to date. Estimated study completion date: September 2021.	Phase 2, 3.

Δ^9^-THC, delta-9-tetrahydrocannabinol; HR, heart rate; BP, blood pressure; CBD, cannabidiol; CO, cardiac output; HDL, high-density lipoprotein; LDL, low-density lipoprotein; TAV, total atheroma volume; CIMT, carotid intima-media thickness; MI, myocardial infarction; ICU, intensive care unit.

## Data Availability

Data sharing not applicable.
